# Supplementary material for: Multi-state detection and spatial addressing in a microscope for ultracold molecules
Source: Nat Commun. 2025 Dec 9;17:518. doi: 10.1038/s41467-025-67212-7 (PMC12804730; doi:10.1038/s41467-025-67212-7)
Supplement: Supplementary file 1 — Supplementary Information [file 41467_2025_67212_MOESM1_ESM.pdf]

Supplemental Material

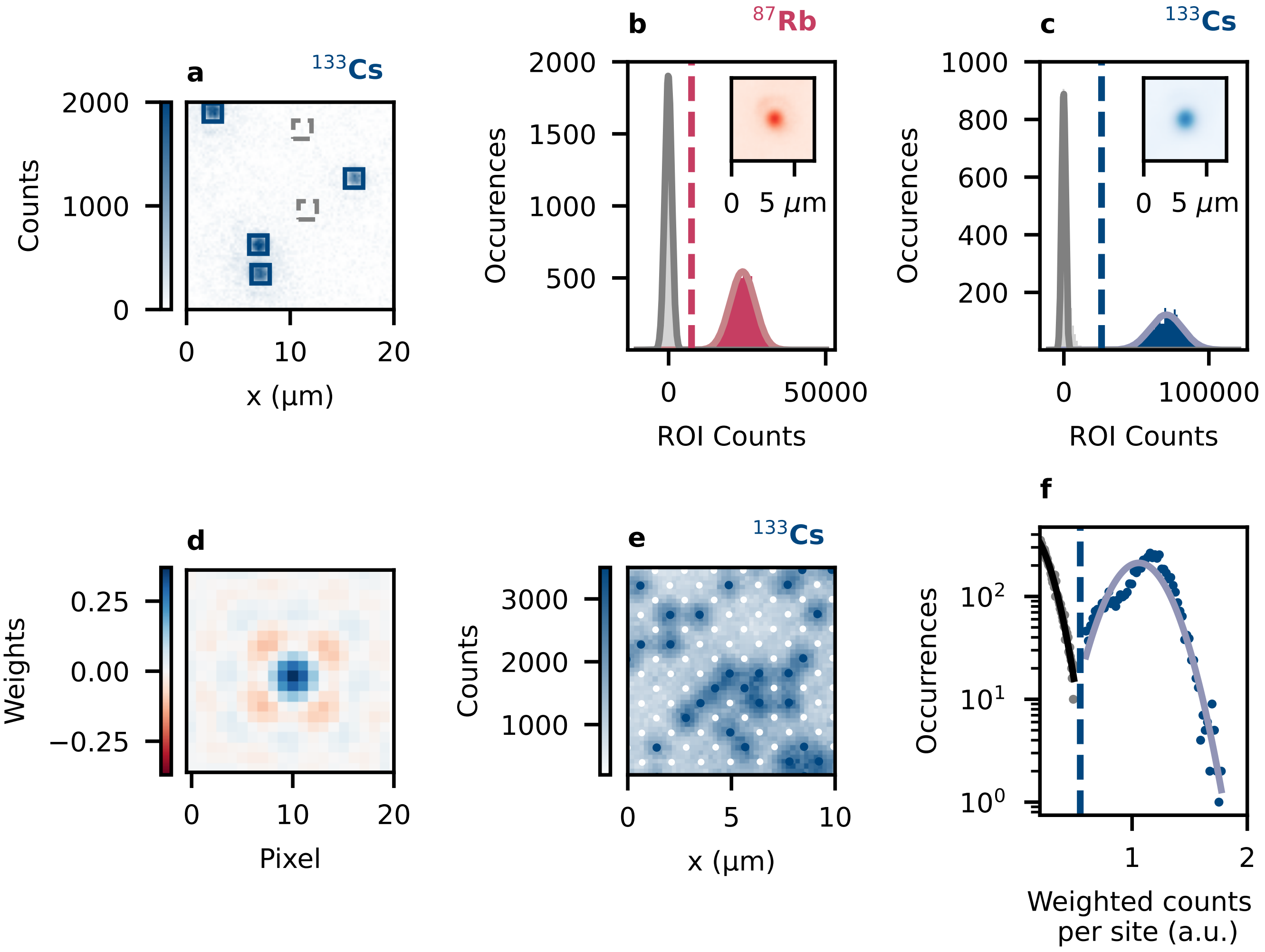

Supplementary Figure 1. **Characterisation of single atom imaging.** **a** Zoom of an exemplary sparse image used to determine the signal-to-noise ratio when imaging isolated atoms. We compare 8x8 pixel regions of interest (ROIs) centred on an atom with ROIs centred in a blank portion of the image. **b** and **c** Histograms of ROI counts to quantify single atom detection fidelity for Rb and Cs, respectively. We observe a clear distinction between the ROIs centred on atoms (red and blue histograms) and those centred on an empty portion of the lattice (grey histograms). The Gaussian fits are used to determine the signal-to-noise ratio reported in the Methods. The measured atomic point spread functions are shown in the insets, with fitted $1/e^2$ radii of 1.19(2) µm for Rb and 1.35(6) µm for Cs. **d** Weights used in the single layer neural network deconvolution algorithm. **e** Example reconstruction of the lattice occupancies for Cs atoms obtained by dissociating RbCs molecules (data corresponds to the shortest hold time in Fig. 2 c). **f** Histogram of the weighted counts per site, i.e. the output of the neural network. We use this to estimate the fidelity of discrimination between empty and filled sites. From the overlap of the Gaussian fits we estimate the error rate is less than 5%.

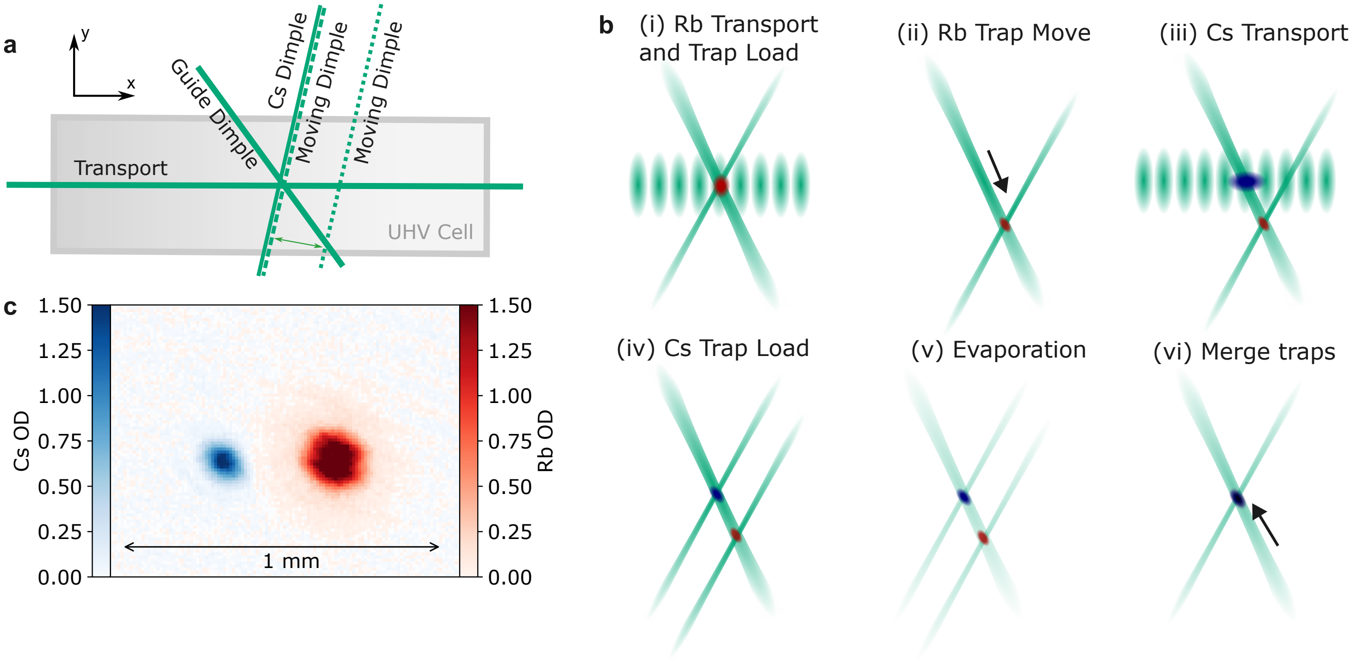

Supplementary Figure 2. **Experimental setup and sequence used for the preparation of a dual-species mixture of Rb and Cs.** **a** The layout of the beams used for optical trapping in the science cell. **b** The sequence used to sequentially load and evaporatively cool Rb and Cs atomic clouds. Rb is first transported to the science cell and then loaded into a crossed optical dipole trap (xODT) formed by the Guide Dimple beam and the Moving Dimple beam. The Rb cloud is moved by translating the position of the Moving Dimple beam. Cs is then transported to the science cell and loaded into an xODT formed by the Guide Dimple beam and the Cs Dimple beam. The two clouds are evaporatively cooled simultaneously before the Rb cloud is moved back to overlap with the Cs cloud, and molecules are prepared. **c** Image of dual-species BECs of Rb and Cs following evaporative cooling in the separated traps.
